# Ink-bottle Effect and Pore Size Distribution of Cementitious Materials Identified by Pressurization–Depressurization Cycling Mercury Intrusion Porosimetry

**DOI:** 10.3390/ma12091454

**Published:** 2019-05-05

**Authors:** Yong Zhang, Bin Yang, Zhengxian Yang, Guang Ye

**Affiliations:** 1 Fujian Provincial University Research Center for Advanced Civil Engineering Materials, Fuzhou University, Fuzhou 350116, China; y.zhang-1@tudelft.nl; 2 College of Civil Engineering, Fuzhou University, Fuzhou 350116, China; 3 Microlab, Section of Materials and Environment, Department of 3MD, Faculty of Civil Engineering and Geosciences, Delft University of Technology, Stevinweg 1, 2628 CN Delft, The Netherlands; g.ye@tudelft.nl; 4 Chengdu Design & Research Institute of Building Materials Industry Co., Ltd, Chengdu 610051, China; b2016yang@126.com

**Keywords:** cementitious, mercury porosimetry, pressurization–depressurization, ink-bottle, pore size distribution

## Abstract

Capturing the long-term performance of concrete must be underpinned by a detailed understanding of the pore structure. Mercury intrusion porosimetry (MIP) is a widely used technique for pore structure characterization. However, it has been proven inappropriate to measure the pore size distribution of cementitious materials due to the ink-bottle effect. MIP with cyclic pressurization–depressurization can overcome the ink-bottle effect and enables a distinction between large (ink-bottle) pores and small (throat) pores. In this paper, pressurization–depressurization cycling mercury intrusion porosimetry (PDC-MIP) is adopted to characterize the pore structure in a range of cementitious pastes cured from 28 to 370 days. The results indicate that PDC-MIP provides a more accurate estimation of the pore size distribution in cementitious pastes than the standard MIP. Bimodal pore size distributions can be obtained by performing PDC-MIP measurements on cementitious pastes, regardless of the age. Water–binder ratio, fly ash and limestone powder have considerable influences on the formation of capillary pores ranging from 0.01 to 0.5 µm.

## 1. Introduction

It has long been considered that microstructural characteristics, which govern almost all the physical and chemical processes taking place in concrete, play a decisive role in the mechanical and durability properties of concrete. Research on this topic has been the focus of considerable research effort, with special attention being paid to determine the pore structure. The alteration of water–binder ratio (w/b) or an addition of blended materials including fly ash and limestone powder will inevitably affect the pore structure of concrete because of the changes of particle packing and chemical composition as well as the changes to the hydration process. A variety of pore size types from a few nanometers to tens of micrometers are created with the process of cement hydration. The main hydration products, calcium-silicate-hydrate C-S-H, consist of molecular-scale interlayer pore spaces as well as nanometric gel pores between C-S-H particles [1]. The micrometric capillary pores are interstitial spaces between unhydrated grains, usually irregular in shape, and represent the originally water-filled space [2]. Air voids, from several micrometers to millimeters in size and spherical in shape, can be naturally or artificially introduced and are randomly distributed in cementitious materials. Pore size characterizations are of great interest for gaining insight to the concrete properties such as strength, shrinkage, self-healing, sorption behavior, alkali-aggregate reaction, frost damage, mass transport, and so on [3,4,5,6,7,8,9].

Mercury intrusion porosimetry (MIP) is one of the most classical techniques for measuring the pore structure of porous systems. It was first proposed by Washburn [10] and then applied by Ritter and Drake [11]. With the assumption that pores in cementitious materials are cylindrical and entirely and equally accessible to mercury, the correlation between the applied pressure *P* (MPa) and the pore diameter *d* (µm) can be described using the Washburn Equation (1):

(1) d=(−4γcosθ)/P,

where *γ* is the surface tension of mercury (N/m); *θ* is the contact angle between mercury and pore wall (°).

At around 1960s, hydraulic pressurized instruments have become commercially available. Since then MIP technique has been rapidly developed. Although there are some perceived fundamental limitations and assumptions, the application of MIP is still regarded as most common and useful for pore structure measurements. Its popularity can be attributed to many aspects. The MIP technique can identify a broad range of pore sizes, which almost cover the whole range of sizes that must be analysed in cementitious materials. It can also figure out a wide variety of other microstructural parameters such as porosity, surface area, density of solid skeleton, etc. These parameters have been proved to correlate well with the macro performances of concrete.

Despite these merits, questions do arise about the reliability of standard MIP measurements [12,13]. MIP suffers from assumptions and drawbacks [14]. The contact angle at pore walls is assumed to be uniform, both advancing angle and receding angle are undifferentiated in usual analysis. Non-cylindrical pores will be identified by the break-through pressure required for mercury to pass through their throats. In the case of poorly connected pore structures, the high pressure applied may break the partitions between non-connected but adjacent pores. The pore structure itself during MIP measurements is assumed to be constant. The pores above threshold diameter cannot be probed.

Among these defects, the accessibility problem, also known as the ink-bottle effect, has been widely recognized so that MIP would overestimate the smaller pore population. There have been considerable debates against the validity of MIP-derived pore size distribution [12,15]. The inconsistency of intrusion-extrusion cycle indicates some mercury has been irreversibly entrapped in the pore space. Due to the accessibility problem, it is suggested that the distribution curve from MIP should be judged as the pore throat size distribution [16].

**Figure 1:**
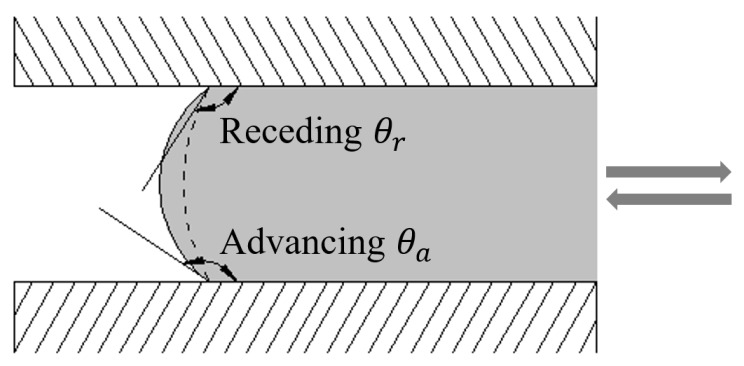
Hysteresis of contact angle between advancing $\theta_{a}$ and receding $\theta_{r}$ in a capillary pore.

Lowell and Shields [17] divided the total intruded mercury into two categories: (i) mercury that can freely cycle into and out of the porous network as the cycle of pressurization-depressurization; (ii) mercury that is irreversibly entrapped in the pores and never extrudes. The hysteresis during intrusion-extrusion cycle is a universal feature of MIP technique, and almost exists in all cementitious materials. Even during a blank test without specimen, intrusion-extrusion cycle shows great hysteresis [16]. The most common explanations are the differences in contact angles between pressurization and depressurization. In a pressurization–depressurization cycle, mercury exhibits different advancing contact angle $\theta_{a}$ and receding contact angle $\theta_{r}$ as shown in Figure 1.

**Figure 2:**
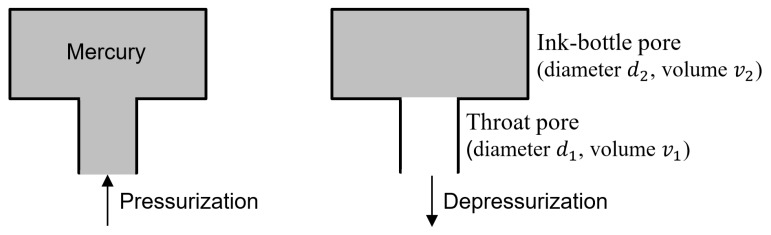
Mercury pressurization-depressurization hysteresis in a pore system with a throat pore connecting an ink-bottle pore.

In the case of pressurization (Figure 2), mercury enters into a pore cavity at the pressure that is determined by the opening neck entrance instead of the actual cavity size. The pore neck is noted as throat pore. Once the intrusion pressure enforces mercury filling the throat pore (diameter *d*_1_), the inner wide size pore (diameter *d*_2_) will be filled simultaneously. The inner wide pore is notably called the ink-bottle pore. On the contrary for depressurization (Figure 2), the mercury in the throat pore *d*_1_ can be freely extruded, while the mercury in the wide body of inner ink-bottle pore *d*_2_ cannot be extruded through the throat pore.

Numerous publications have described the pore structure features of hydrating/hydrated cementitious materials by MIP tests [11,12,13,14,15,16,17,18,19,20,21,22,23,24]. The intrudable porosity, as well as the threshold diameter can reasonably be determined with a high accuracy. However, the standard MIP cannot provide valid estimates of pore size distribution because it measures pore size on the basis of the diameter of accessible throat pores through which the mercury penetrates the microstructure to reach internal pores. The large (ink-bottle) pore can be filled with mercury only after the small (throat) pore has been intruded.

By making use of the pressurization–depressurization hysteresis, Zhou et al. [18] investigated the volumes of throat pores and ink-bottle pores. Instead of the standard MIP test protocol, the applied pressure is increased from the minimum to the maximum by repeating pressurization–depressurization cycles, whereby the ink-bottle pore volume corresponding to each throat pore can be determined. In order to analyze the pore size distribution, it has been assumed that the fractions of the ink-bottle pores are equal to that of the pores with the diameter larger than the corresponding throat pores [18]. Based on volume fractions, each ink-bottle pore volume will be allocated to the larger pore sizes. This novel method employs the principles of pressurization–depressurization cycles and is capable of better understanding the pore size distributions.

This paper aims to afford deeper insights into the pore size features of the cementitious system by adopting the pressurization–depressurization cycling mercury intrusion porosimetry (PDC-MIP). Paste specimens were prepared and cured at ages from 28 to 370 days. The effects of binder type (OPC, fly ash and limestone powder) and w/b (0.4, 0.5 and 0.6) are analysed and discussed.

## 2. Principles of Pressurization-Depressurization Cycling Mercury Intrusion Porosimetry (PDC-MIP)

### 2.1. Test Sequence

**Figure 3:**
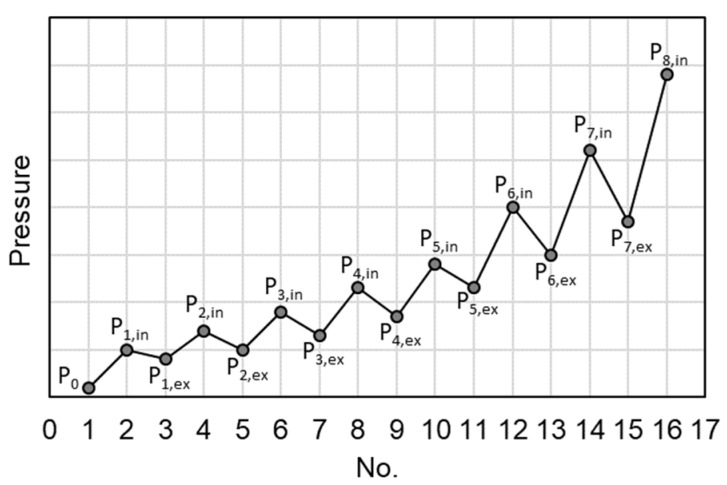
Illustration of the pressurization-depressurization cycling mercury intrusion porosimetry (PDC-MIP) test sequence.

PDC-MIP test is conducted with two stages: a low-pressure stage and a high-pressure stage (comprising a number of pressurization-depressurization cycles at increasing pressures from the minimum to the maximum pressure). As illustrated in Figure 3, the pressure applied starts from $P_{0}$, increasing up to $P_{1,\mathrm{in}}$ and then decreasing to $P_{1,\mathrm{ex}}$ for the first pressurization-depressurization cycle. $P_{1,\mathrm{in}}/P_{1,\mathrm{ex}}$ is set equal to the ratio between the cosine values of advancing contact angle and receding contact angle $\cos\theta_{a}/\cos\theta_{r}$. In the nth cycle, the pressure increases from $P_{n-1,\mathrm{ex}}$ to $P_{n,\mathrm{in}}$ and then decreases to $P_{n,\mathrm{ex}}$. The newly intruded volume and extruded volume of mercury in the nth cycle are recorded as $V_{n}^{\mathrm{in}}$ and $V_{n}^{\mathrm{ex}}$, respectively. The test is finalized when the maximum pressure is applied. For more details about the principles of the PDC-MIP test, reference can be made to previous reports [18,23].

Following this test sequence, the incremental volume of mercury intruding at pressures from $P_{n-1,\mathrm{in}}$ to $P_{n,\mathrm{in}}$ can be divided into two parts: throat pore volume $V_{n}^{\mathrm{th}}$ and ink-bottle pore volume $V_{n}^{\mathrm{ink}}$. Both values can be calculated as follows:

(2a) $$V_{n}^{\mathrm{th}}=V_{n}^{\mathrm{ex}},$$

(2b) $$V_{n}^{\mathrm{ink}}=V_{n}^{\mathrm{in}}-V_{n}^{\mathrm{ex}},$$

### 2.2. Pore size Analysis

An assumption is introduced that at the nth pressurization-depressurization cycle, the size distribution of ink-bottle pores is equal to that of the pores with diameter larger than the corresponding throat pores, i.e., from *d*_1_ to *d*_n−1_. According to the volume fractions of each throat pore size, the ink-bottle pore volume $V_{n}^{\mathrm{ink}}$ is reallocated to the pores with diameter from *d*_1_ to *d*_n−1_. Then the pore size distribution of the specimen can be computed step-by-step as shown below:

Step 1: assuming the largest pore does not have ink-bottle effect:

(3) $$V_{1,1}=V_{1}^{\mathrm{th}}=V_{1}^{\mathrm{in}},$$

where $V_{1,1}$ is the pore volume at diameter *d*_1_ calculated at Step 1.

Step 2: the ink-bottle volume $V_{2}^{\mathrm{ink}}$ is allocated to pores with diameter larger than *d*_2_ (i.e., *d*_1_).

(4a) $$V_{1,2}=V_{1,1}+V_{2}^{\mathrm{ink}},$$

(4b) $$V_{2,2}=V_{2}^{\mathrm{th}}=V_{2}^{\mathrm{ex}},$$

where $V_{1,2}$ and $V_{2,2}$ are the volumes of pores with diameters of *d*_1_ and *d*_2_, respectively, calculated at Step 2.

Step 3: the ink-bottle volume $V_{3}^{\mathrm{ink}}$ is allocated to pores with diameter larger than *d*_3_ (i.e., *d*_1_ and *d*_2_).

(5a) $$V_{1,3}=V_{1,2}+V_{3}^{\mathrm{ink}}\left(\frac{V_{1,2}}{V_{1,2}+V_{2,2}}\right),$$

(5b) $$V_{2,3}=V_{2,2}+V_{3}^{\mathrm{ink}}\left(\frac{V_{2,2}}{V_{1,2}+V_{2,2}}\right),$$

(5c) $$V_{3,3}=V_{3}^{\mathrm{th}}=V_{3}^{\mathrm{ex}},$$

where $V_{1,3}$, $V_{2,3}$ and $V_{3,3}$ are the volumes of pores with diameters of *d*_1_, *d*_2_ and *d*_3_, respectively, calculated at Step 3.

Step *n*: similar as previous steps:

(6a) $$V_{n,n}=V_{n}^{\mathrm{th}}=V_{n}^{\mathrm{ex}},$$

(6b) $$V_{i,n}=V_{i,n-1}+V_{n}^{\mathrm{ink}}\left(\frac{V_{i,n-1}}{V_{1,n-1}+V_{2,n-2}+\cdots V_{n-1,n-1}}\right),$$

where $V_{i,n}$ indicates the volume of pores size *d_i_* calculated at step *n*.

**Figure 4:**
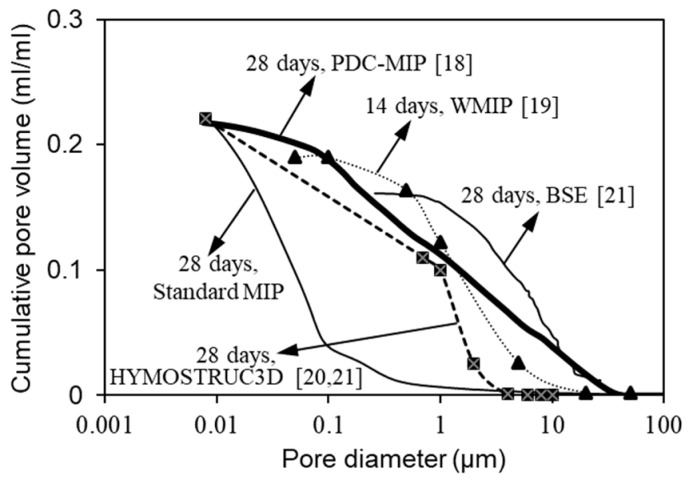
Comparison of pore size distribution between standard MIP, PDC-MIP, WMIP (wood’s metal intrusion porosimetry), BSE (backscattered electron image) and HYMOSTRUC3D (hydration, morphology and structure 3-dimensional formation).

In order to validate the efficiency of the PDC-MIP approach, the pore size results derived from other methods including the WMIP (wood’s metal intrusion porosimetry) [19], HYMOSTRUC3D (hydration, morphology and structure 3-dimensional formation) [20,21] and BSE (backscattered electron image) [21] are used for a comparison. As indicated in Figure 4, the PDC-MIP yields a relatively coarser pore size distribution compared with the standard MIP, but that the pore size distribution from PDC-MIP agrees well with that obtained from WMIP, BSE and HYMOSTRUC3D. It is reasonable to consider that PDC-MIP can substantially reduce the impact of accessibility problem and it provides a more reliable estimate of pore sizes than the standard MIP.

## 3. Experimental Program

### 3.1. Raw Materials

Given that the paste matrix contributes most to the porosity of concrete, this paper focuses on analysing the pore structure of paste specimens. The raw materials used in this work were CEM I 42.5N ordinary Portland cement (OPC), fly ash (FA) and limestone powder (LP). The mean particle size, determined by laser diffraction, was 24.1, 25.0 and 33.5 µm for OPC, FA and LP, respectively. A range of paste mixtures was designed. The details of the mix compositions are shown in Table 1.

### 3.2. Sample Preparation

Cement pastes with w/b of 0.4, 0.5, and 0.6 were mixed in a HOBART mixer at low speed for 1 min and at high speed for 2 min. Next the fresh paste was poured into plastic bottles and shaken continuously on the vibration table to remove big air bubbles and sealed with lids thereafter. In order to avoid bleeding, the paste samples were rotated for one day before placing them in a humid room with the condition of 20 ± 0.2 °C and 98% RH. At the age of 28, 105, 182 and 370 days, the paste samples were taken out of the plastic bottles and then split into small pieces (around 1 cm^3^). Liquid nitrogen was used to stop the further hydration of the cement pastes [21]. These pieces were moved into a freeze-dryer with temperature of −24 °C and under vacuum at 0.1 Pa. After the water loss was below 0.01% per day, the paste specimens could be used for PDC-MIP measurements.

### 3.3. Pore Structure Characterization by PDC-MIP Test

PDC-MIP measurements were performed with Micromeritics PoreSizer 9320 (Micromeritics, Norcross, GA, USA), which has a capacity of 210 MPa, corresponding the measurable minimum pore diameter of 0.007 µm. The test started with a continuous pressurization from 0.004 to 0.15 MPa (low pressure run), then eighty pressurization–depressurization cycles were repeated at increasing pressures from 0.15 to 210 MPa (high pressure run). Volume measurements were corrected by a blank run without specimen. Due to the rough texture of the exterior of samples and air bubbles lying at the surface, errors may exist in the mercury intrusion during low pressure run and this volume is not of interest in this study. The contact angles corresponding to pressurization and depressurization are 138° and 128°, respectively [18,24]. An equilibration time of 60 s was used for each pressurization or depressurization step. At the end of PDC-MIP measurements, the specimens showed no visible cracks after an examination under an optical microcopy at ×100. All porosity and pore size results presented in this paper are based on the average of three parallel measurements.

## 4. Results and Discussion

### 4.1. Reproducibility of PDC-MIP Measurements

Five replicate paste samples of OPC paste with w/b = 0.5 and cured after 28 days were employed to check the reproducibility of PDC-MIP measurements. A variety of microstructural parameters obtained by PDC-MIP are summarized in Table 2. For an overview of these group data, the sample weight is around 5 g. The difference in total porosity between the maximum and the minimum value is 0.87% with an average standard deviation of 0.32%. The standard deviations for other parameters, i.e., bulk density and skeletal density, are all less than 2%. In comparison to standard MIP measurements, the PDC-MIP measurements exhibit a very similar reproducibility in characterizing these main microstructural parameters.

### 4.2. Distribution of Throat Pores and Ink-Bottle Pores

**Figure 5:**
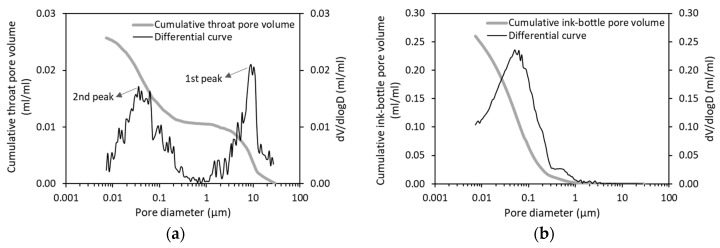
Distribution of (**a**) throat pores and (**b**) ink-bottle pores in OPC paste (w/b = 0.5, 28 days) determined by PDC-MIP measurements.

Figure 5a presents the cumulative throat pore volume and its differential curve for OPC paste with w/b of 0.5 determined by means of PDC-MIP measurements. Obviously, the throat pore volume increases with decreasing pore diameter. Two bumps corresponding to the 1st peak and the 2nd peak are observed. Accordingly, the pore sizes can be divided into three parts: >1 µm, 0.3–1 µm and <0.3 µm. Of particular interest is that only a small amount of throat pores in the range of 0.3–1 µm is present in the cement paste.

Figure 5b shows the distribution of ink-bottle pores as a function of pore diameter. One main peak is observed, which is different from the distribution of throat pores. Most of the ink-bottle pore volumes are associated with the throat pores below 0.3 µm. A comparison between Figure 5a,b indicates that the total ink-bottle pore volume is about ten times larger than the total throat pore volume. It is worthwhile to note that the ink-bottle volume obtained by PDC-MIP differs from that obtained by standard MIP because the two measurements are very different in the test sequence. Far more important note is that irrespective of the maximum pressure applied, a pressurization-depressurization cycle always results in an ink-bottle volume. This finding holds for all the paste mixtures tested in this study, suggesting that the ink-bottle effect exists in the entire pore size range of cementitious materials. The ink-bottle pores are constricted predominantly by the throat pores below 1 µm in size (see Figure 5b).

#### 4.2.1. Effect of w/b

**Figure 6:**
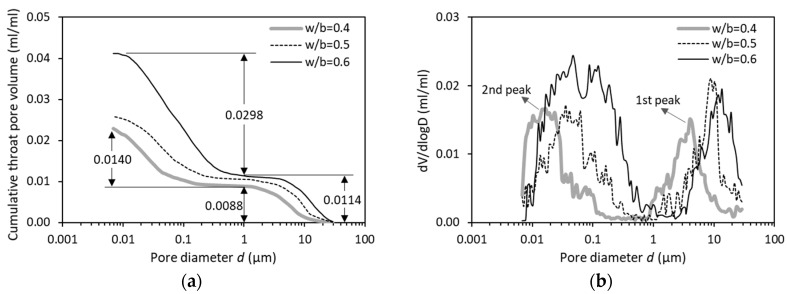
(**a**) Distribution of throat pores and (**b**) differential curves for OPC pastes (28 days) with w/b of 0.4, 0.5 and 0.6.

Figure 6a reports the cumulative throat pore volume of OPC paste with w/b of 0.4, 0.5 and 0.6. It is obvious that a higher w/c leads to a higher total amount of throat pores. A closer observation of the curves shows that by increasing the w/b from 0.4 to 0.6, the volume of throat pores at *d* > 1 µm increases slightly from 0.0088 to 0.0114 while the volume of throat pores at *d* ≤ 1 µm increases tremendously from 0.0140 to 0.0298. This finding points to a significant impact of w/b on the formation of small pores (*d* ≤ 1 µm) in hydrating cementitious systems. Figure 6b shows the differential curve of the throat pore size distribution. As can be seen, both the first and the second peak shift towards to a coarser pore sizes for a higher w/b. The pore size range included in the 2nd peak is broadened from 0.007–0.1 µm to 0.007–0.6 µm when increasing the w/b from 0.4 to 0.6.

**Figure 7:**
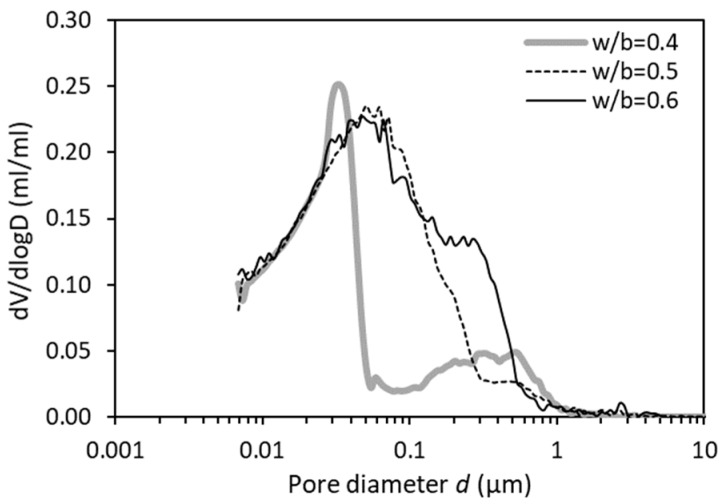
Differential curves of ink-bottle pore size distribution for OPC pastes (28 days) with w/b of 0.4, 0.5 and 0.6.

Figure 7 provides the distribution of ink-bottle pore volume as a function of pore diameter. Regardless of the w/b from 0.4 to 0.6, the curves at *d* < 0.025 µm are almost overlapped, indicating that the ink-bottle effect changes little in these very fine pore sizes range. Between w/b = 0.5 and w/b = 0.6, the main peaks are still very similar (except some changes for large pores at *d* > 0.12 µm). Both curve patterns, however, differ substantially from that of w/b = 0.4. It seems that the influence of the w/b on the ink-bottle effect becomes particularly pronounced for low w/b (e.g., 0.4).

#### 4.2.2. Effect of Fly Ash (FA)/Limestone Powder (LP)

**Figure 8:**
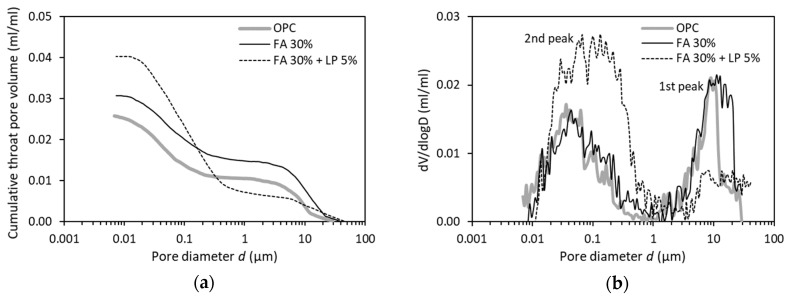
Effect of fly ash and/or LP on (**a**) distribution of throat pores and (**b**) differential curves in paste specimens (w/b = 0.5, 28 days).

Figure 8a gives the cumulative throat pore volume in three cement pastes with different binders but with the same w/b of 0.5. Compared with the neat OPC binder, the binary binder (FA 30%) shows similar pattern (but higher) of cumulative throat pore volume vs. pore diameter. However, with the further addition of LP, the ternary binder (FA 30% + LP 5%) exhibits a tremendously different pattern. Figure 8b gives the differential curves of the results presented in Figure 8a. It is clear that the presence of FA 30% primarily results in a volume change of the large pores (above 13 µm) as compared to the OPC binder. Far more interesting is that the addition of a minor amount of LP 5% remarkably alters the distribution of throat pores, with the throat pores above 1 µm considerably decreased and the throat pores below 1 µm significantly increased. It appears that the addition of FA and/or LP does not change the pore size range included in the second peak, a different observation from that caused by altering w/b as already shown in Figure 6b.

**Figure 9:**
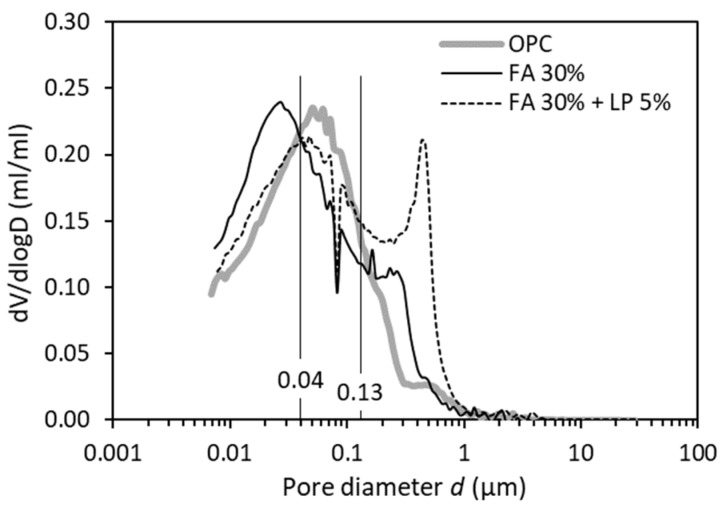
Differential curves of ink-bottle pore size distribution for paste specimens with different binders (w/b = 0.5, 28 days).

As indicated in Figure 9, the binary binder (FA 30%) has a larger ink-bottle effect at *d* < 0.04 µm than the neat OPC binder. The ternary binder (FA 30% + LP 5%) has a similar ink-bottle effect at *d* < 0.13 µm but a much larger ink-bottle effect at *d* ≥ 0.13 µm, compared to the neat OPC binder.

### 4.3. Evolution of Pore Size Distribution with Age

#### 4.3.1. Pore Sizes Characterized by Standard MIP

**Figure 10:**
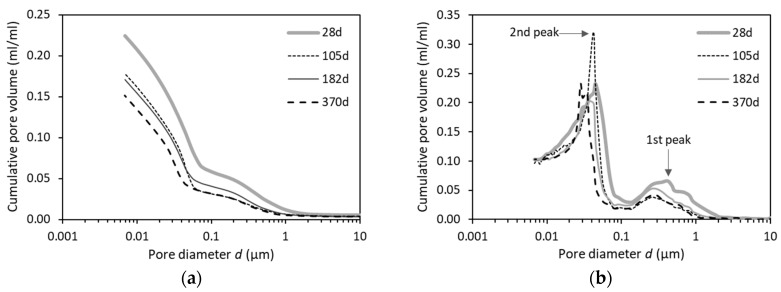
(**a**) Pore size distributions and (**b**) differential curves in OPC pastes (w/b = 0.4) with different ages characterized by standard MIP measurements.

**Figure 11:**
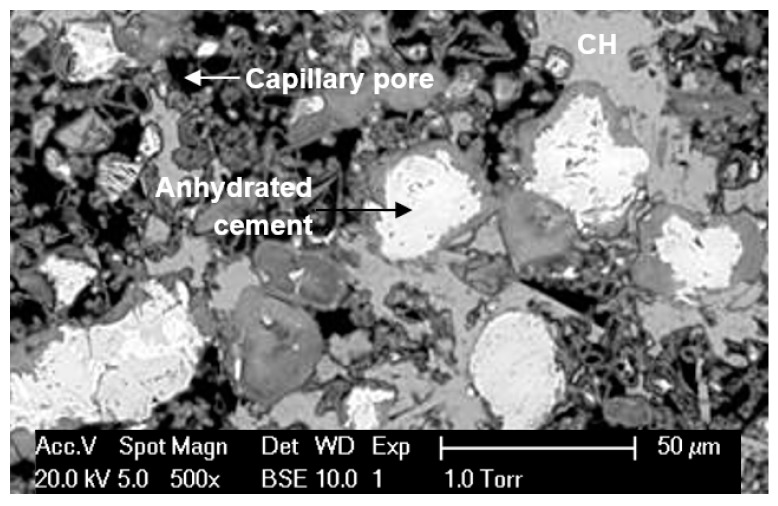
BSE image of the microstructure of OPC paste (w/b = 0.4, 28 days) [21].

Figure 10a shows the experimental results of the accessible pore volume of OPC pastes (w/b = 0.4) filled by mercury in standard MIP measurements. All the curves were based on the average of three measurements, with a good accuracy (±5%). The general trend is as expected that the total pore volume decreases with a higher curing age. The total pore volume decreases greatly in the first 105 days. Afterwards, it evolves limited up to half a year, indicating a dormant period to have taken place during which little cement has reacted. This observation agrees well with the findings as reported by Luke and Glasser [25]. Two peaks can be observed in the differential pore size distribution curves, as shown in Figure 10b. The first peak is quite low, and the majority of the porosity is contributed to by the small pores located in the second peak (i.e., <0.1 µm). However, a considerable number of large pores (>0.1 µm) are present in cement paste, as can be proved by the backscattered image shown in Figure 11 that the dark area represents the large capillary pores (>0.1 µm) [21]. Undoubtedly, the large pores derived by standard MIP measurements have been remarkedly underestimated, which is attributable to the ink-bottle effect.

#### 4.3.2. Pore Sizes Characterized by PDC-MIP

**Figure 12:**
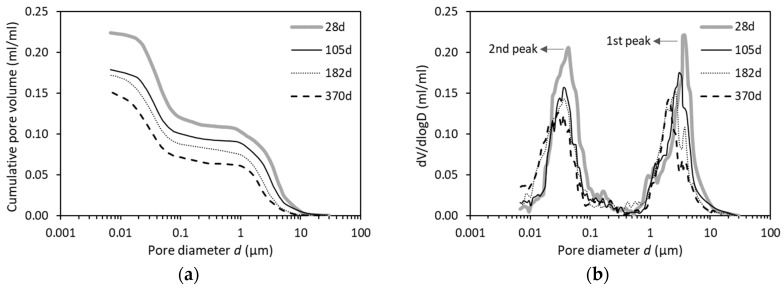
(**a**) Pore size distributions and (**b**) differential curves in OPC pastes (w/b = 0.4) with different ages characterized by PDC-MIP measurements.

Based on the PDC-MIP test sequence and pore size analysis, as earlier noted in Section 2, the pore size distributions of various paste mixtures were determined. Figure 12a presents the results of the pore size distribution of OPC paste with w/b of 0.4 at different ages ranging from 28 to 370 days. Figure 12b depicts the corresponding derivative pore size distribution in logarithmic plot.

The pore size distribution curves (Figure 12a) shift to the left with increasing age, that is, the pore structure becomes increasingly finer. Nevertheless, the overall shape of the distribution curves is observed to exhibit very similar pattern and can be divided into three parts. With a decrease of the pore diameter, the cumulative pore volume starts with a rapid rise, followed by a plateau evolution, then ended with another abrupt increase. This phenomenon can also be reflected by the results given in Figure 12b that the bimodal distributions are observed. The 1st bump covers mainly in the pore size range of 0.8–10 µm, with the pore diameter corresponding to the top of the first peak (i.e., first critical pore diameter) decreasing from around 4 µm at 28 days to approximately 2 µm at 370 days. The second bump primarily covers the pore sizes between 0.01–0.1 µm, but that the 2nd critical pore diameter changes little with age from 28 to 370 days.

The coexistence of the first and second peaks have been confirmed previously by researchers such as Diamond [12], Ye [21], Cook [26], etc. They have carried out a large number of experimental measurements based on a variety of techniques. They maintained the idea that the two peaks in the pore size distribution curve can be attributed to the two different mechanisms of pore structure formation. One is the capillary pore system with diameter larger than 0.1 µm, the other is the gel pore system with diameter smaller than 0.1 µm. It has been shown that standard MIP measurements of early age paste specimens (e.g., seven days) enable to obtain a pore size distribution curve with two bumps. One bump corresponds to capillary pore system and another corresponds to gel pore system. While at later ages, i.e., after 28 days, the bump corresponding to capillary pore system becomes weak or even disappears as indicated by standard MIP [21].

It is noteworthy that the disappearance of the first peak for capillary pore system in standard MIP tests should not be attributed to the absence of large capillary pores but is rather owed to the inherent accessibility problem (ink-bottle effect) of the MIP test. According to the Powers model [2], there are two types of capillary pores: *small capillary pores* that represent the void space due to insufficient packing of hydration products and *large capillary pores* that refer to the void space out of hydration products. At an early age, all the capillary pore space is fully interconnected. As ongoing cement hydration, the large capillary pores are gradually clogged or segmented by hydration products, and become partially connected or even completely disconnected due to the continuous precipitations of hydrates. During mercury penetration, the small capillary pores act as neck entrance to large capillary pores. The large capillary pores cannot be penetrated with mercury only when their neighbouring connected small capillary pores have been intruded. As a consequence, nearly all the volume of large capillary pores (capillary pore system) cannot be reflected by standard MIP test. In contrast, the PDC-MIP approach is able to create a bimodal curve and the large capillary pores (>0.1 µm) can still be clearly identified even after one year’s cement hydration. The two bumps in the distribution of capillary pores by PDC-MIP are consistent with the two different kinds of capillary pores as defined by Powers [2].

By studying the pore size distribution of OPC paste at different ages, the PDC-MIP tests are proved to provide a more realistic pore sizes as compared to standard MIP tests.

### 4.4. Effects of w/b and Fly Ash on Pore Size Distribution

**Figure 13:**
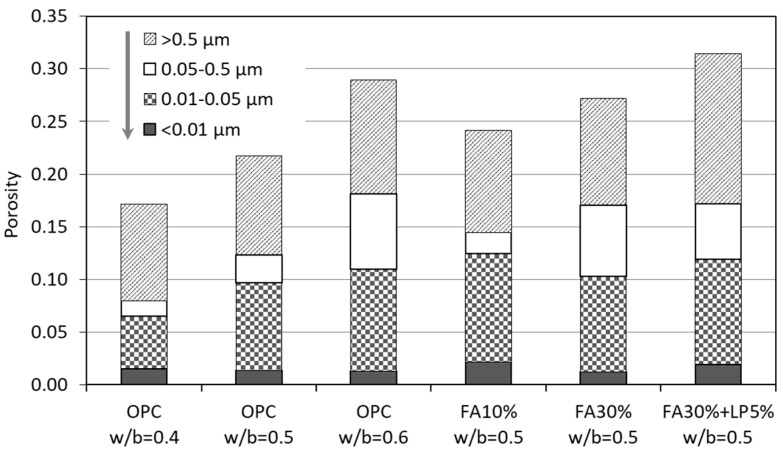
Pore size distribution of various cement pastes at 182 days obtained by PDC-MIP tests.

Pore size distribution analyses were carried out for a wide range of pore diameter from 0.007 to 10 µm based on PDC-MIP measurements. The measured results were divided into four pore size groups: gel pores (< 0.01 µm), small capillary pores (0.01–0.05 µm), medium capillary pores (0.05–0.5 µm) and large capillary pores (>0.5 µm), according to Sidney and Young [27]. Figure 13 shows the details of the pore size distribution of six specimens at the age of 182 days.

For OPC pastes with increasing w/b from 0.4 to 0.6, as shown in Figure 13, the volumes of both gel pores (<0.01 µm) and large capillary pores (>0.5 µm) change marginally. In contrast, the small capillary pores (0.01–0.05 µm) show an obvious increase and particularly the medium capillary pores (0.05–0.5 µm) increase substantially. These findings reveal that the dilution effect (caused by increasing water-to-cement ratio) mainly works on the capillary pores in the range of 0.01–0.5 µm. Compared to the neat OPC paste (w/b = 0.5), the addition of FA 10% almost doubles the volume of gel pores (<0.01 µm) but halves the volume of medium capillary pores (0.05–0.5 µm), in the meantime the volume of small capillary pores (0.01–0.05 µm) is increased while the volume of large capillary pores (>0.5 µm) changes little. This is a typical observation for blended materials with smaller pore size resulting from pozzolanic reactions. By increasing the replacement level of fly ash from 10% to 30%, the medium capillary pores (0.05–0.5 µm) rise significantly while the small capillary pores (0.01–0.05 µm) are reduced. A further addition of LP 5% greatly dilutes the cementitious system, reflected by a much higher total porosity. To be specific, the ternary binder (FA 30% + LP 5%) has a higher amount of gel pores, small and large capillary pores only that the amount of medium capillary pores is less, compared to the binary binder (FA 30%). All these provide evidence that the w/b or FA or LP plays an important role in the formation of small and medium capillary pores, i.e., 0.01–0.5 µm. A similar observation about the effect of w/b on pore sizes has been reported previously by Farzanian and Ghahremaninezhad [28].

**Figure 14:**
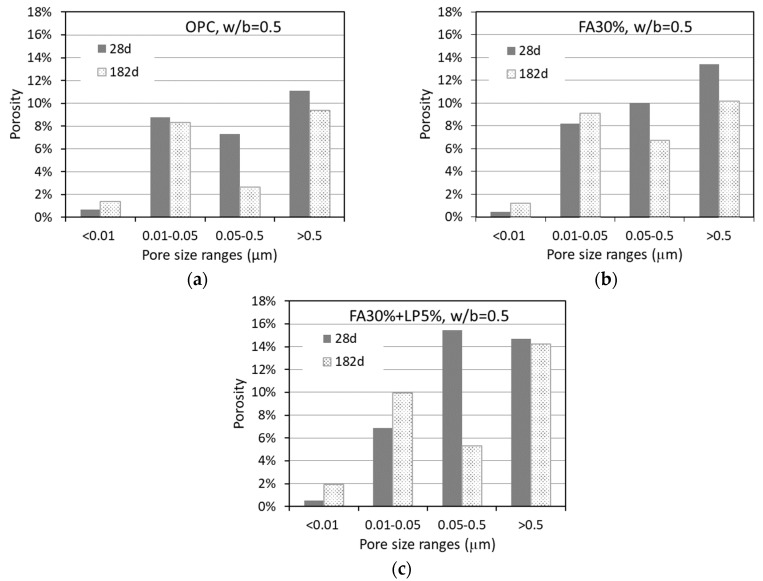
Volume changes of pores in different size range with hydration from 28 to 182 days for (**a**) OPC, (**b**) binary and (**c**) ternary pastes.

Figure 14a–c displays the changes of pore size distribution resulting from continuous hydration in the paste specimens with OPC binder, binary binder (FA 30%) and ternary binder (FA30% + LP 5%). As indicated, the pore sizes move towards to a finer distribution as the hydration proceeds from 28 to 182 days. The volume of gel pores (<0.01 µm) increases while the volume of capillary pores (>0.01 µm) is considerably decreased. In particular, the gel pores of FA-blended pastes (FA 30%, FA 30% + LP 5%) are significantly increased with age from 28 to 182 days, indicating the pozzolanic reactions of FA particles occurred in later hydration periods. As a result, the calcium hydroxide is transformed into secondary C-S-H gel, whose effect is to refine the pore structure so that the volume of capillary pores is decreased [29,30,31]. Interestingly, with continuous hydration, the small capillary pores (0.01–0.05 µm) are decreased in OPC paste (due to continuous cement hydration) while increased in FA-blended pastes (due to pozzolanic reactions resulting in transformation of large pores to small pores). It seems that the presence of LP (FA 30% + LP 5%) can promote the production of gel pores compared with the system without LP (FA 30%).

## 5. Conclusions

An alternative measurement procedure, viz. PDC-MIP, is described and utilized to characterize the ink-bottle effect and pore size distribution for a range of cementitious pastes. PDC-MIP measurements enable to understand the distribution of throat pores and ink-bottle pores. Compared to standard MIP, the PDC-MIP provides a more reliable estimation of pore sizes. This research work provides new insights into the pore sizes and pore connections in cementitious systems.

From the experimental results presented earlier in this paper, key findings are the following:

(1) Water-binder-ratio (w/b) influences the throat pore volume primarily by affecting the amount of throat pores below 1 µm. The addition of fly ash increases the volume of throat pores larger than 13 µm. Incorporating limestone powder leads to a considerable rise in throat pores below 1 µm while a drastic drop in throat pores above 1 µm.
(2) The ink-bottle effect is demonstrated over the entire range of pore sizes in cementitious pastes.
(3) Bimodal pore size distributions are obtained by performing PDC-MIP on cementitious pastes, regardless of the age (up to one year). The first peak corresponds to the capillary pore system and the second peak is created by the gel pore system.
(4) With cement hydration process from 28 to 370 days, the pore sizes of cement paste shift towards to a finer distribution. The critical pore diameter corresponding to the first peak clearly decreases with age while the critical pore diameter of the second peak changes little.
(5) Altering the w/b or adding fly ash/limestone powder can significantly affect the volume of capillary pores in the range of 0.01–0.5 µm.

## Figures and Tables

**Table 1:** Mix proportions of paste samples (weight percentage) with ordinary Portland cement (OPC), fly ash (FA) and limestone powder (LP).

Mixtures	Water-Binder-Ratio (w/b)	OPC	FA	LP
M1	0.4	100%		
M2	0.5	100%		
M3	0.6	100%		
M4	0.5	90%	10%	
M5	0.5	70%	30%	
M6	0.5	65%	30%	5%

**Table 2:** Data of five replicates for OPC paste (w/b = 0.5, 28 days) by PDC-MIP.

Sample No.	Weight (g)	Total Porosity (%)	Bulk Density (g/mL)	Skeletal Density (g/mL)
1	5.0590	27.95	1.5256	2.0948
2	4.9223	27.99	1.5433	2.1315
3	4.8298	27.88	1.5287	2.1403
4	5.1023	28.75	1.5144	2.0922
5	5.5869	28.13	1.5325	2.1256
Average	5.1001	28.14	1.5289	2.1169
Standard deviation	0.2620	0.32	0.0093	0.0197
